# Identifying Genetic Predisposition to Dozer Lamb Syndrome: A Semi-Lethal Muscle Weakness Disease in Sheep

**DOI:** 10.3390/genes16010083

**Published:** 2025-01-14

**Authors:** Morgan R. Stegemiller, Margaret A. Highland, Kathleen M. Ewert, Holly Neaton, David S. Biller, Brenda M. Murdoch

**Affiliations:** 1 Department of Animal, Veterinary and Food Science, University of Idaho, Moscow, ID 83844, USA; 2 WI Veterinary Diagnostic Laboratory, University of Wisconsin-Madison, Madison, WI 54812, USA; 3 Notkwyta Ranch, Inc., McLouth, KS 66054, USA; 4 Neaton Polypays, Watertown, MN 55388, USA; 5 Department of Clinical Sciences, Kanas State University, Manhattan, KS 66506, USA

**Keywords:** WGS, GWAS, dozer lamb syndrome, CELF1

## Abstract

Background: Lamb health is crucial for producers; however, the percentage of lambs that die before weaning is still 15–20%. One factor that can contribute to lamb deaths is congenital diseases. A novel semi-lethal disease has been identified in newborn Polypay lambs and termed dozer lamb syndrome. This study aims to determine if there is a genetic predisposition to dozer lamb syndrome. These lambs are weak and unable to lift their heads, suckle, and swallow, resulting in nasal reflux. Methods: Genetic analyses, including a genome-wide association, runs of homozygosity, and fine mapping to determine haploblock within regions of interest, were utilized in determining genetic predispositions to dozer lamb syndrome. Results: The genome-wide association study identified a region of chromosome 15 with three significant SNPs (*p*-values of 6.81 × 10^−6^, 5.71 × 10^−6^, and 8.52 × 10^−6^). Genetic analysis identified a run of homozygosity on the same region of chromosome 15 with an odds ratio of 236.7. Fine mapping of this region identified three haploblocks associated with the dozer lamb syndrome (*p*-value = 2.41 × 10^−5^). Conclusions: The most significant and promising gene in this region is *CELF1*, which is known to play an important role in muscle development. Abnormal CELF1 abundance and cellular location are reported to result in abnormal muscle development. Identification of genetic aberrations associated with dozer lamb syndrome provides a tool for decreasing or eliminating the genotype and, thus, the associated phenotype(s) from Polypay sheep.

## 1. Introduction

Lamb health is vital to producers for sustaining flock productivity across the sheep industry. Approximately 15–20% of lambs die before weaning, and the majority of this loss occurs within three days of birth [1]. These deaths can be caused by multiple factors, including dystocia, infectious disease, extreme weather, nutrition, and congenital defects. Management practices such as providing shelter during lambing and winter feeding of pregnant ewes have helped decrease lamb mortality [2].

One of the most beneficial changes a producer can use is breeding strategies to select against congenital defects and increase lamb production and health. One such defect is congenital myopathies, genetic muscle disorders that often present as muscle weakness and lack of muscle tone at birth [3]. Myotonic diseases have been identified in many species, including humans, dogs, horses, goats, and pigs, with many different genes and genetic variants associated with myopathies. As the identification of genetic causes of muscular dystrophies in animals has increased, so has the utility of utilizing these animals as models to study diseases in humans [4,5,6,7,8].

While the use of next-generation sequencing has increased the identification of genetic mutations, there are still challenges in identifying the causes of congenital myopathies [9]. One challenge is when the severity of the condition can fluctuate between individuals with the same genetic variant [10]. Another challenge is that myogenesis is a complex biological process, and mutations in different pathways may result in similar pathologic features [11]. To continue to increase our knowledge, it is important to study congenital myopathies across multiple species in which they have been identified to determine genetic variants that may result in the disruption of “normal” myogenesis. Understanding the genetic underpinning of the biological mechanisms behind normal and abnormal muscle development and growth can aid in our understanding of muscle development, as well as provide knowledge to develop strategies to prevent occurrences.

A novel semi-lethal disease, termed “dozer lamb syndrome,” has been identified in Polypay lambs in the United States and, to the best of our knowledge, is being described here for the first time [12]. The aim of this study is to characterize the morphology of lambs with dozer lamb syndrome and determine if there is a genetic predisposition to this syndrome.

## 2. Materials and Methods

### 2.1. Animals and Samples

**Figure 1:**
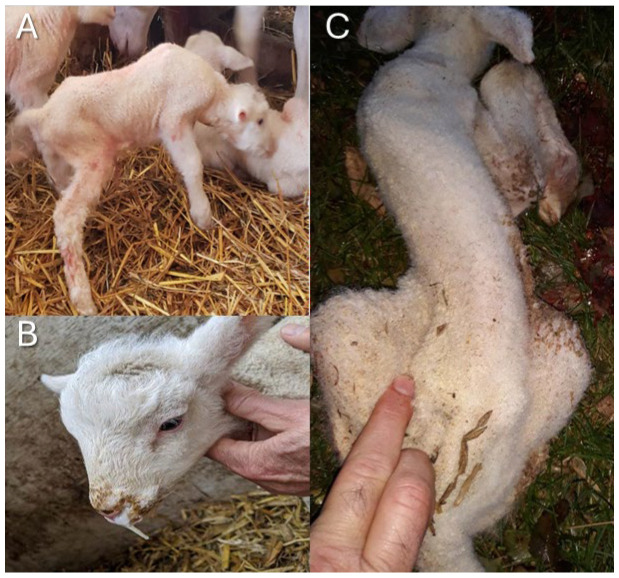
The physical signs present in some lambs reported with dozer lamb syndrome. (**A**). Difficulty standing and unable to lift head above the level of the back. (**B**). Nasal reflux without the presence of a cleft palate. (**C**). Scoliosis.

The American Polypay Sheep Association asked producers to examine their flocks for any lambs that were born with symptoms consistent with the dozer lamb syndrome profile and to monitor the breed for this problem. Affected Polypay newborn lambs have difficulty lifting their heads (Figure 1A), struggle to suckle and swallow, and often have nasal reflux of milk in the absence of a cleft palate (Figure 1B). Phenotypic characteristics can include a short neck and ears, curvature or a dip in the spine (scoliosis or kyphosis), and contracted front legs (Figure 1C and Video S1). Lambs often perish from malnutrition, develop aspiration pneumonia, or are euthanized due to failure to thrive. Samples described below were collected by the producers as a part of routine maintenance or by veterinarians, so institutional approval was not necessary. Docked tail tissue and/or blood in EDTA tubes from suspected affected lambs, and closely related relatives (parents, siblings, half-siblings, and parents of half-siblings) were sent to the University of Idaho. A total of 14 dozer lamb syndrome cases and 287 control, 32 related samples, and 255 previously published Polypay were used in this study.

### 2.2. DNA Extraction and Genotyping

DNA was extracted from samples at the University of Idaho using a phenol-chloroform method as previously described [13]. Samples were genotyped on either the Applied BiosystemTM AxiomTM Ovine genotyping 50k array (Thermo Fisher Scientific, San Diego, CA, USA) or the AgResearch Sheep Genomic 60K SNP chip (GenomNZ, AgResearch, Lincoln, New Zealand). The Polypay sheep samples obtained from the public domain had been genotyped on either the Ovine SNP 50 BeadChip or the Affymetrix SNP chip (Illumina Inc., San Diego, CA, USA) [14,15]. There were 41,771 SNPs present on all three chips that also passed quality thresholds of call rate > 0.9, minor allele frequency > 0.01, and Hardy-Weinberg Equilibrium > 1 × 10^−6^ and were used for subsequent analyses.

### 2.3. Genome-Wide Association Study

A genome-wide association study was conducted using SNPs on the 26 autosomes to identify genotypes associated with the dozer lamb syndrome. A logistic regression model was run with a recessive model matching the mode of inheritance using Plink v1.9 [16]. Logistic regression models are used to analyze the relationship between two data factors, usually yes or no, such as in a case/control data set. A lambda adjustment of 2.1 was used to correct for population stratification present in the sample set. a significant value threshold was designated at an adjusted −log10(*p*-Value) > 5. Regions of interest (ROIs) were determined as 50 kb upstream and downstream of significant GWAS SNPs.

### 2.4. Runs of Homozygosity

DetectRuns in R studio was used to analyze the genotypes for runs of homozygosity (ROH) to identify regions that could be harboring deleterious recessive alleles [17]. Parameters for identifying ROH included using sliding windows with a window size of 15 SNPs, max opposite and missing window at 1 SNP, maximum gap of 10 Mb, minimum length of 10 kb, and minimum density of 1 SNP/10 kb. An odds ratio was calculated using a 2 × 2 contingency table to test for significance in analyzing ROH identified in the dozer lambs [18].

### 2.5. Sequence Data Used for Fine Mapping

Seven affected dozer lambs and four unaffected parents were whole genome sequenced using the Illumina platform at 2 × 150 bp to a minimum depth of 10× coverage and an average depth of 12×. Ten unrelated Polypay with whole genome sequence (WGS) available in the public domain were used as control samples. These 21 samples were mapped to ARS-UI_Ramb_v2.0 using bwa-mem [19,20]. Variants were identified as a cohort using Freebayes and then filtered to retain variants with a phred quality score of 20 [21]. Fine mapping from the WGS was used to analyze the regions of interest determined from the GWAS. Identified SNPs in the ROI were filtered for a call rate of 0.98 and analyzed in Haploview v4.2 to determine haplotype blocks [22]. Chi-square tests were also conducted in Haploview on each haplotype block, and R-squared linkage disequilibrium values were reported. Significant SNPs and INDELs from these regions were examined in JBrowse and NCBI for annotated genes [23]. A literature review was conducted on the annotated genes in the ROIs and examined for known biological functions that influence muscle development or other symptoms that correspond to the dozer lamb phenotype.

## 3. Results

### 3.1. Genome Wide Association Study

**Figure 2:**
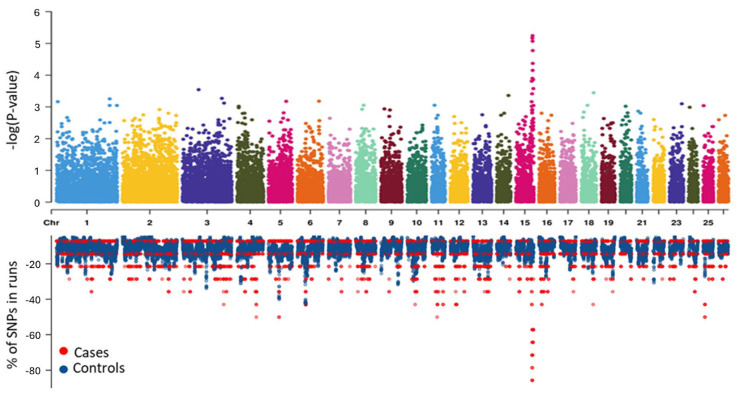
Miami plot illustrating the comparison of the GWAS (**top**) and percentage of SNPs in runs of homozygosity (ROH) (**bottom**) analyses of dozer lamb cases and unaffected control sheep.

A recessive logistic GWAS test was conducted to test for potential genetic associations with dozer lamb syndrome. This analysis revealed three significant SNPs identified on chromosome 15. The results from the GWAS are illustrated in Figure 2, and *p*-values of significant SNPs and genes within 50 kb are reported in Table 1. The second most significant SNP (rs407879401) had a *p*-value of 5.71 × 10^−6^. We considered 50 kb up and downstream of the SNP and the two annotated genes within that region as ROI 1. The first (rs628219689) and third (rs608876284) significant SNPs (*p*-values = 6.81 × 10^−6^ and 8.53 × 10^−6^ respectively) were 64 kb apart and had an r-squared linkage disequilibrium (LD) value of 0.74. Given the amount of linkage between these two SNPs, we considered 50 kb upstream of rs608876284 and 50 kb downstream of rs628219689 as ROI 2. This region spans 164 kb and contains eight annotated genes.

### 3.2. Runs of Homozygosity

Runs of Homozygosity (ROH) were analyzed using the 50K SNP data and were used to interrogate regions associated with dozer lamb syndrome. One ROH was identified as significantly associated with dozer lamb syndrome with an odds ratio of 236.7. This significant ROH included 34 SNPs and spanned a region of 1.99 Mb on chromosome 15 from 74.55–76.54 Mb. In analyzing this ROH, a smaller subset region of the ROH from 76.22–76.54 Mb was identified with an increased odds ratio of 347.1. This smaller subset consisted of six consecutive SNPs and spanned a 328 kb region. In addition, the ROH subset contained nine annotated genes, *CELF1*, *PTPMT1*, *NDUFS3*, *FAM180B*, *C1QTNF4*, *MTCH2*, *AGBL2*, *FNBP4*, and *NUP160*. Both the ROH and GWAS analyses indicated that the same region on chromosome 15 was significantly associated with dozer lamb syndrome (Figure 2 and Figure S1).

### 3.3. Whole Genome Fine Mapping

**Figure 3:**
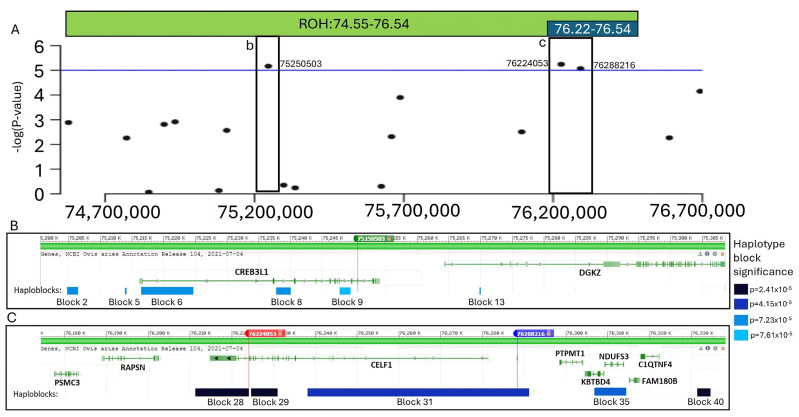
The region of significance on chromosome 15, the significant results identified by the GWAS and ROH analyses, and the significant haplotype blocks are identified from fine mapping. (**A**). The significant GWAS results on chromosome 15 with the location of the ROH are identified in the green bar, and the subset of the ROH is indicated with the blue bar. The black framed boxes (**b**) and (**c**) show the locations of the regions of interest in relation to the GWAS and ROH results. (**B**). Region of interest 1: 50 kb on either side of the SNP rs407879401 with the boxes identifying the significant haploblocks in the region of interest in relation to the genes and significant SNP. (**C**). Region of interest 2: the 164 kb surrounding SNPs rs628219689 and rs608876284 with the boxes identifying the significant haploblocks in the region of interest in relation to the genes and significant SNP.

The subset of samples was whole genome sequenced, and the data were used to fine-map the two regions of interest (ROI 1 and ROI 2). Using the SNPs identified from the whole genome sequence, a total of 41 haploblocks were identified, with 25 haploblocks in ROI 1 and 16 haploblocks in ROI 2 (Table S1). Significant haploblocks were determined as haploblocks with a *p*-value ≤ 7.61 × 10^−5^. Six Significant haploblocks were determined as haploblocks with a *p*-value -values for each haplotype in the three most significant haploblocks are shown in Table 2, Table 3 and Table 4. The significant haploblocks and genes in the ROIs, in conjunction with the results of the GWAS and ROH, are shown in Figure 3.

**Figure 4:**
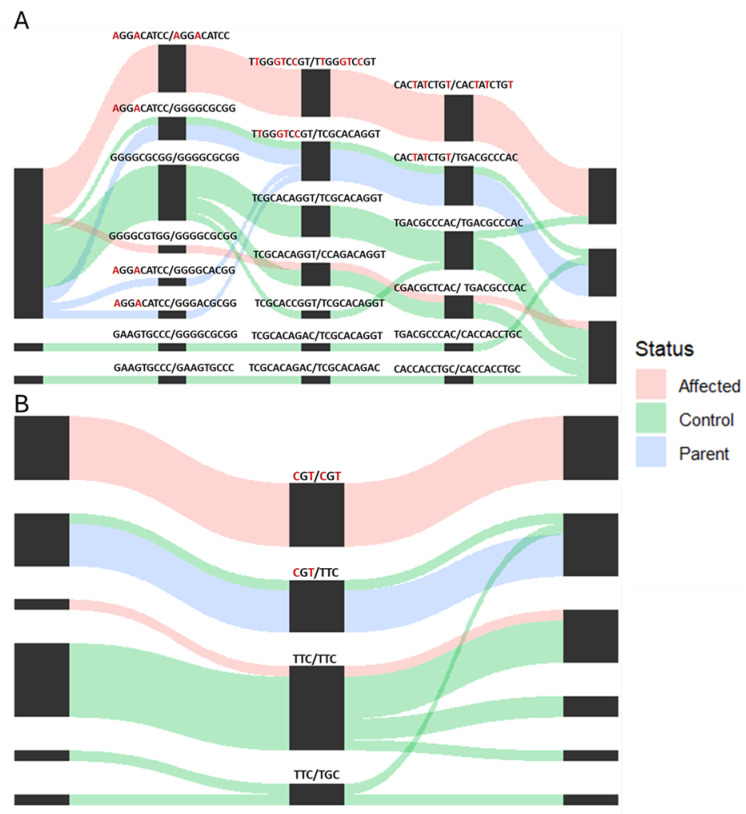
Diplotype distribution for (**A**). haploblock 28 and (**B**). haploblock 29 for the affected, control, and parental samples. The black boxes show the segments present in each haploblock, and the SNPs in red are SNPs present only in the affected haplotype.

The significant haploblocks were examined using the sample diplotypes and matched haplotype pairs (Figure 4). In all three of the most significant haploblocks, six of the seven dozer lambs were homozygous for the affected haplotype. One of the dozer lambs included in the whole genome sequence did not contain any of the affected haplotypes and may potentially be a phenocopy of the dozer lamb disease. All the parental samples contain one copy of the affected haplotype and one normal haplotype, as expected, with a recessive model of inheritance observed in the GWAS. One control sample from the unrelated sheep had one copy of the affected haplotype and one normal haplotype in the top three haploblocks. Haploblock 28, which is located in the 3′ utr region of *CELF1* (Figure 4), consists of 43 SNPs; of these, nine homozygous SNP alleles are present only in the affected haplotype. Haploblock 29 is located at the end of *CELF1*, and of the 22 SNPs in the block, only three were present in the haplotype of affected animals (Figure 4). Haploblock 40 contains 5 SNPs, one of which is present only in the affected haplotype (Table 4). The three most significant haploblocks all have a total of 13 SNPs that were only present in the affected haplotype.

## 4. Discussion

Analyzing the lambs submitted with dozer lamb syndrome showed no gross pathological defects. Although there is the potential of a nutritional deficiency causing these effects, six of the affected lambs identified had a twin/triplet, with a total of 10 twins/triplets reported as normal. If this disease were caused by a nutrient deficiency, we would expect to see a larger number of twins/triplets affected. In addition, three normal twins/triplets contain the ROH and are homozygous for the affected alleles at the significant SNPs. This would imply that the penetrance of dozer lamb syndrome is incomplete and, taken with the varying degrees of severity reported from producers, could indicate varying expressivity in this disease [24].

The results of ROH and GWAS analyses, as well as fine mapping using WGS, identified a region on chromosome 15 that is significantly associated with dozer lamb syndrome. Two regions of interest were identified from 50 kb on either side of the significant SNPs, with ROI 2 being 164 kb and consisting of both rs628219689 and rs608876284. The genes in these regions of interest were examined to potentially identify known biological functions that could influence the dozer lamb syndrome phenotype.

The most promising positional candidate gene is the CUGBP Elav-like family member 1 (*CELF1*). This gene contained the most significant SNP as well as the two most significant haplotype blocks. In addition, *CELF1* is located within the smaller and most significant ROH, consisting of six SNPs. CELF1 is a shuttling protein that can regulate gene splicing and editing in both the nucleus and cytoplasm [25]. CELF1 is a binding protein that regulates pre-mRNA alternative splicing and, through this, plays a role in mRNA editing and translation and early embryonic development of heart and skeletal muscle [26,27]. During heart development, CELF1 promotes the inclusion of exon 5 in the cardiac Tropin T protein, but as development occurs, the low level of CELF1 promotes the exclusion of exon 5 in the adult human heart [27].

CELF1 has been reported to play a role in myotonic dystrophy type 1 (DM1), a type of muscular dystrophy in humans, via interactions with the dystrophia myotonica-protein kinase (*DMPK*) gene [28]. DM1 has been associated with progressive muscle weakness as affected individuals age; however, congenital DM1 is more severe and can lead to early lethality, ranging from death within the first year of life to reduced survival beyond the mid-30s [29,30,31]. DM1 patients have increased satellite cell numbers that fail to undergo terminal differentiation, especially in congenital cases [32,33]. Although the primary genetic cause of DM1 has been associated with a trinucleotide repeat in *DMPK*, overexpression of *CELF1* has also been identified as being highly associated with DM1 [28,34]. Analysis of individuals with the genetic mutation responsible for DM1 identified hyperphosphorylation of nuclear CELF1 in conjunction with overexpression [35].

Previous research has analyzed the effects of CELF1 on muscular development without the presence of the *DMPK* repeat. Studies have shown that overexpression of *CELF1* without any other genetic change is reported to cause a significant drop in myotube formation and impairment of terminal myocyte differentiation due to defective cell cycle exit, believed to be caused by RNA toxicity [36,37]. However, myotube formation was accelerated in CELF1 knockdown cells, resulting in increased precocious myotube formation [37]. Research showed that the location of CELF1 plays an important role as overexpression of CELF1 in the nucleus leads to moderate histologic defects, such as myofiber variability, hyperpigmentation, myofiber necrosis, and severe myopathy, while overexpression of cytoplasmic CELF1 presented only minor myofiber variability [26,38]. The 3′ and 5′ utrs have vital roles in the subcellular localization of CELF1, and it has been reported that a nuclear localization signal exists on the C terminus of CELFs [39,40]. There are five distinct 5′ utr and four 3′ utr isoforms in *CELF1* in humans, and these isoforms influence the cellular localization of CELF1 [41,42]. Haploblock 28, the most significant haploblock (*p* = 5.41 × 10^−5^), is located in the 3′ utr and contains 9 SNPs present only in the affected haplotype. Although the exact role of the different *CELF1* isoforms and their subcellular location and role in muscle development has yet to be demonstrated, genetic variants in these regions are significantly associated with the dozer lamb disease. These studies show the vital role of *CELF1* expression levels for proper muscle growth and differentiation and how the genetic variants associated with the dozer lamb syndrome could impact the muscle development of the dozer lambs.

Although *CELF1* is the most significant candidate gene associated with dozer lamb, six genes in the ROIs with functions could also contribute to the dozer lamb phenotype. Receptor-associated protein of the synapse, *RAPSN*, is involved in the transmission of signals from the nerve cell to the muscle and has been associated with congenital myasthenic syndrome, which is indicated by hypotonia and muscle weakness in the skeletal muscles of the face and limbs of affected individuals [43,44,45,46]. However, no variants nor haplotype blocks are associated with dozer lamb syndrome identified in the RAPSN gene. Two genes in the ROIs are associated with muscle function: cAMP-responsive element binding protein 3 (*CREB3L1*) and diacylglycerol kinase zeta (*DGKZ*). CREB3L1 is a transcription factor that is highly expressed in neurons and is hypothesized to upregulate the *NTRK2* gene to promote vascular smooth muscle cell-neuron interactions [47]. DGKZ has been associated with mechanical stimulation of muscle hypertrophy through stimulation of the mTOR pathway [48]. To the best of our knowledge, this gene has not been associated with any muscle histological abnormalities. Three genes have known mitochondrial functions: *NDUFS3*, *PTPMT1*, and *PSMC3*. NADH-ubiquinone oxidoreductase (*NDUFS3*) has been associated with mitochondrial disease and muscle degeneration, but knockout *Ndufs3* mice had increased central nuclei in their muscle fibers and did not have the same physical characteristics as identified in the dozer lambs [49]. PTPMT1 functions in the mitochondria to dephosphorylate phosphatidylinositol phosphates, which impacts pyruvate utilization as a mitochondrial energy source [50]. Mice with specific skeletal and heart *Ptpmt1* knocked out were indistinguishable from littermates up to three months of age, suggesting that the knockout does not affect heart or muscle development [50]. Proteosome 26S subunit ATPase 3 (*PSMC3*) is an ATPase subunit involved in the ATP-dependent degradation of ubiquitinated proteins but has not been reported to be associated with muscle disease [51]. Although these genes are within the ROIs and could play a role in the dozer lamb syndrome, the previous literature does not identify similar effects that are identified in the dozer lambs.

## 5. Conclusions

This study used genetic information to reveal a significant association between the region on chromosome 15 and dozer lamb syndrome based on 50K ROH, GWAS, and haplotypes identified from fine mapping. The most promising gene in the significant region is CELF1, as this gene has the first and third most significant GWAS SNPs and the top two haploblocks. Several studies in humans and mice have associated this gene and changes in its expression levels with proper or myotonic muscle development. It should be noted that the diagnosis of this disease was challenging and complex, with a limited number of lambs identified as affected in this study. While the genetic analyses revealed significant haploblocks and a strong association with chromosome 15: 76.17–76.34 Mb, a specific causal mutation was not able to be identified. An increase in the number of affected lambs would increase the power of this study and the effort toward identifying the causal mutation. The significant SNP identified in this study, rs628219689, was added to Flock54 in order to provide producers with a second test to monitor the prevalence of the associated affected allele in their flocks and continue to evaluate for additional dozer lambs that might be born. We aim to monitor the situation using this data and validate our findings with any future dozer lambs reported.

## Figures and Tables

**Table 1:** The significant SNPs locations, regions of interest (ROIs), and genes within the ROIs.

Chr	Position (bp)	rs Number	*p*-Value	Region of Interest	Genes Within the ROI
15	75,250,503	rs407879401	6.81 × 10^−6^	ROI 1: 75,200,503–75,300,503	*CREB3L1*, *DGKZ*
15	76,224,053	rs628219689	5.71 × 10^−6^	ROI 2: 76,174,053–76,338,216	*PSMC3*, *RAPSN*, *CELF1*, *PTPMT1*, *KBTBD4*, *NDUFS3*, *FAM180B*, *C1QTNF4*
15	76,288,216	rs608876284	8.52 × 10^−6^		

**Table 2:** The Frequencies and *p*-values of each haplotype in haploblock 28, chromosome 15: 76,211,571–76,224,239.

Haplotype	Segment 1						Segment 2									Segment 3										Segment 4										Segment 5								Affected Frequency	Control Frequency	Chi-Square *p*-Value
1	G	T	C	A	C	A	A	G	G	A	C	A	T	C	C	T	T	G	G	G	T	C	C	G	T	C	A	C	T	A	T	C	T	G	T	G	C	A	G	C	G	T	A	0.857	0.179	2.41 × 10^−5^
2	G	T	C	A	C	A	G	G	G	G	C	G	C	G	G	T	C	G	C	A	C	A	G	G	T	T	G	A	C	G	C	C	C	A	C	A	T	C	A	A	A	C	T	0.071	0.536	0.0035
3	G	T	C	A	C	A	G	G	G	G	C	G	T	G	G	C	C	A	G	A	C	A	G	G	T	T	G	A	C	G	C	C	C	A	C	A	T	C	A	A	A	C	T	0.071	0.000	0.1523
4	A	C	T	G	T	T	G	A	A	G	T	G	C	C	C	T	C	G	C	A	C	A	G	A	C	C	A	C	C	A	C	C	T	G	C	G	C	A	G	C	G	T	A	0.000	0.107	0.2037
5	G	T	C	A	C	A	G	G	G	G	C	G	C	G	G	C	C	A	G	A	C	A	G	G	T	C	G	A	C	G	C	T	C	A	C	A	T	C	A	A	A	C	T	0.000	0.071	0.3055
6	G	T	C	A	C	A	G	G	G	A	C	G	C	G	G	T	C	G	C	A	C	A	G	G	T	T	G	A	C	G	C	C	C	A	C	A	T	C	A	A	A	C	T	0.000	0.036	0.4742
7	G	T	C	A	C	A	G	G	G	G	C	A	C	G	G	T	C	G	C	A	C	A	G	G	T	T	G	A	C	G	C	C	C	A	C	A	T	C	A	A	A	C	T	0.000	0.036	0.4742
8	G	T	C	A	C	A	G	G	G	G	C	G	C	G	G	T	C	G	C	A	C	C	G	G	T	T	G	A	C	G	C	C	C	A	C	A	T	C	A	A	A	C	T	0.000	0.036	0.4742

**Table 3:** The Frequencies and *p*-values of each haplotype in haploblock 29, chromosome 15: 76,224,530–76,230,703.

Haplotype	Segment 1																Segment 2			Segment 3			Frequency of Affected Haplotypes	Frequency of Control Haplotypes	Chi-Square *p*-Value
1	G	T	A	A	C	C	A	A	A	T	T	T	T	A	T	A	C	G	T	G	C	G	0.857	0.179	2.41 × 10^−5^
2	C	C	G	G	T	T	G	G	G	C	C	C	G	G	C	G	T	T	C	T	T	A	0.071	0.571	0.0019
3	C	C	G	G	T	T	A	G	G	C	C	C	G	G	C	G	T	T	C	T	T	A	0.071	0.000	0.1523
4	C	C	G	G	T	T	G	G	G	C	C	C	G	G	C	G	T	T	C	G	T	A	0.000	0.143	0.1371
5	G	T	A	A	C	C	G	A	A	T	T	T	T	A	T	A	T	G	C	T	C	G	0.000	0.036	0.4742
6	G	T	A	A	C	C	G	A	A	T	T	T	T	A	T	A	T	G	C	G	C	G	0.000	0.036	0.4742
7	G	C	G	G	T	T	G	G	G	C	C	C	G	G	C	G	T	T	C	T	T	A	0.000	0.036	0.4742

**Table 4:** The Frequencies and *p*-values of each haplotype in haploblock 40, chromosome 15: 76,332,583–76,334,724.

Haplotype						Frequency of Affected Haplotypes	Frequency of Control Haplotypes	Chi-Square *p*-Value
1	G	T	G	T	G	0.857	0.179	2.41 × 10^−5^
2	G	C	G	C	G	0.071	0.321	0.0729
3	A	C	A	C	A	0.071	0.250	0.1647
4	A	C	A	C	G	0.000	0.143	0.1371
5	G	C	G	T	G	0.000	0.071	0.3055
6	A	C	G	C	G	0.000	0.036	0.4742

## Data Availability

The data presented in this study are available on request from the corresponding author due to privacy restrictions.

## Supplementary Materials

The following supporting information can be downloaded at: https://www.mdpi.com/article/10.3390/genes16010083/s1, Figure S1: QQ plot for the genome-wide association study analyzing dozer lamb cases and unaffected control sheep. Table S1: Haploblocks identified in regions of interest one and two, block number, location, number of SNPs, and chi-square *p*-value of affected haplotype. Video S1: Video showing a reported dozer lamb struggling to walk and unable to lift its head above the level of the back.
