# Supplementary figures and images for: Identifying Genetic Predisposition to Dozer Lamb Syndrome: A Semi-Lethal Muscle Weakness Disease in Sheep
Source: Genes (Basel). 2025 Jan 14;16(1):83. doi: 10.3390/genes16010083 (PMC11764822; doi:10.3390/genes16010083)

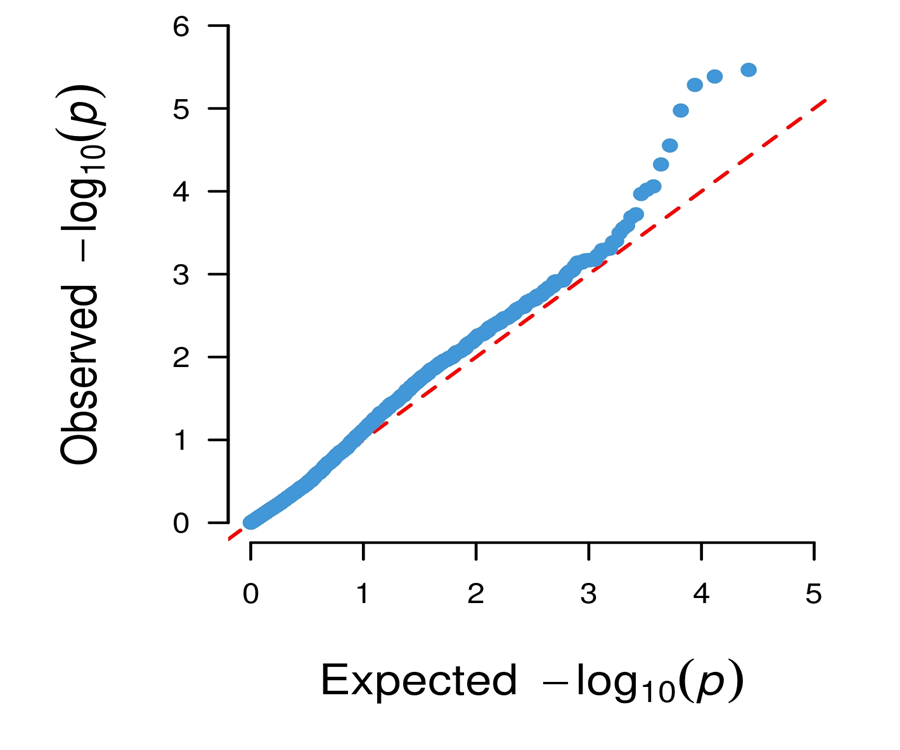

Supplement: Supplementary file 1 [file genes-16-00083-s001.zip › Rhino_Lamb_FigureS1.png]
